# Child disability and family-centred care in East Africa: Perspectives from a workshop with stakeholders and health practitioners

**DOI:** 10.4102/ajod.v11i0.931

**Published:** 2022-07-29

**Authors:** Pauline Samia, Susan Wamithi, Amina Kassam, Melissa Tirkha, Edward Kija, Ayalew Moges, Arnab Seal, Peter Rosenbaum, Robert Armstrong

**Affiliations:** 1 Department of Paediatrics and Child Health, Aga Khan University, Nairobi, Kenya; 2 Brain and Mind Institute, Aga Khan University, Nairobi, Kenya; 3 Department of Paediatrics, Muhimbili University of Health and Allied Sciences, Dar Es Salaam, United Republic of Tanzania; 4 Department of Paediatrics, Debre Tabor Hospital, Debre Tabor, Ethiopia; 5 Department of Paediatrics, Leeds Community Healthcare NHS Trust, Leeds, United Kingdom; 6 Department of Health Research Methods, Evidence and Impact, University of Leeds, Leeds, United Kingdom; 7 Department of Paediatrics, McMaster University, Hamilton, Canada

**Keywords:** cerebral palsy, rehabilitation, quality of life, Africa, family-centred

## Abstract

**Background:**

Our understanding of child disability has undergone major changes over the last three decades transforming our approach to assessment and management. Globally there are significant gaps in the application of these 21st century models of care. There is recognition that economic, cultural, and social factors influence transitions in care and there is need to consider contextual factors.

**Objectives:**

A two-day workshop brought together key stakeholders to discuss current models of care and their application in the East African context. This article summarises workshop proceedings and identifies a broadly supported set of recommendations that serve to set a direction for health professionals, families, family-based disability organisations, communities and government.

**Method:**

Presentations followed by facilitated round-table sessions explored specific themes with participants reporting their responses communally. Future actions were agreed upon by relevant stakeholders.

**Results:**

Many barriers exist to care for children with disabilities in East Africa, including stigma and a lack of human and infrastructural resources. In addition, significant disparities exist with regard to access to medication and specialist care. The International Classification of Functioning framework needs to be translated to clinical practice within East Africa, with due recognition of the importance of family-centred care and emphasis on the life course theory for disability care. Family-centred care, educational initiatives, advocacy on the part of stakeholders and involvement of government policymakers are important avenues to improve outcomes.

**Conclusion:**

Further education and data are needed to inform family-centred care and multidisciplinary team implementation across East African care contexts for children with disabilities.

## Introduction

The World Health Organization (WHO) defines disability as an umbrella term that covers impairments, activity limitations and restrictions in participation (World Health Organization/The World Bank 2011). Disability is not considered a health problem, but rather an interaction between a person’s bodily functions and features of the environments in which they live (Groce 2018; World Health Organization/The World Bank 2011). The United Nations Children’s Fund provides a global estimate of 230 million children, aged 0–17 years, having a disability, with 28.9 million children living in Eastern and Southern Africa (UNICEF 2021). More than 50% of children with disabilities live in rural settings and only 39% of this population attend a primary school (World Health Organization/The World Bank 2011).

Advances in the field of child development and disability create opportunities to impact the life course of children with disabilities, whether the child is living in high or low resource settings. The transition from a traditional treatment model approach to disability to a socio-cultural ability model has transformed the way professionals understand their role in relation to the family and broader societal influences. This transformation is aligned with professionals bringing a higher level of evidence-informed science to the therapeutic interventions that a child with a disability may benefit from at any given stage of their development.

Over the last three decades, two key conceptual shifts have occurred that create the opportunity to have greater impact on the lives of children with a disability. The first one is the fundamental role of the family, not the health professional, in understanding and supporting the development of their child. Family-centred care means that providing effective care for a child includes attention to the immediate social structure within which the child lives and facilitating operations within that context. The child therefore is not managed singly and the unit of care is the family.

Historically the ‘answer’ was seen to be with the professional and hence families were dependent on the guidance of the professional. The growth of family-centred care has occurred through recognition that the family best understands their child and has the greatest investment of time and commitment for their child’s development and that they are key equal partners to the health professionals in understanding the needs of their child and the therapeutic interventions that can have an impact (Makworo, Bwibo & Omoni 2016). Equally important is the recognition that the family themselves are the greatest possible therapeutic intervention and that supporting and empowering families has enormous opportunity to impact their child’s development.

The second important conceptual shift came with the introduction and evolution of the International Classification of Function (ICF) by the World Health Organization in 2001 that shifted language from ‘impairment’, ‘disability’ and ‘handicap’ to neutral terms of ‘body function and structure’, ‘activity’ and ‘participation’ and emphasising an interaction amongst these components that defines the health and well-being of the individual. Disability becomes a generic term that captures the individual in the context of their environment.

**FIGURE 1:**
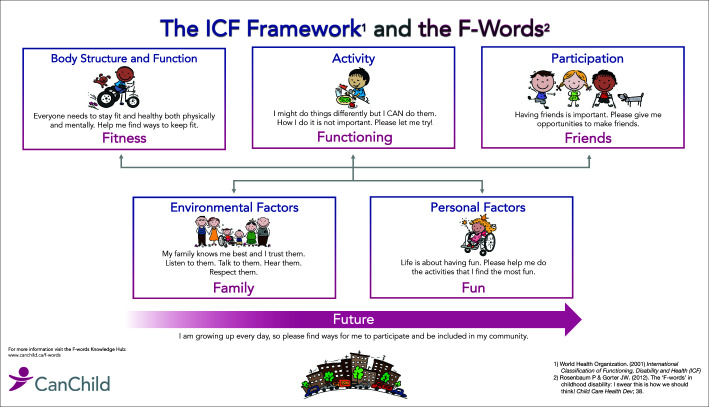
The International Classification of Function framework linked to the F-words.

The ICF is intended to be a framework for classification, but the more powerful impact has been the conceptual shift in how we understand disability and the opportunities for supporting healthy development in children who have a disability. For children, this model has become a highly effective education tool with the development of the ‘F-words’ by the CanChild Centre for Childhood Disability Research (Rosenbaum & Gorter 2012). The ‘F-words’ of ‘fitness’, ‘function’, ‘friendship’, ‘family factors’, ‘future’, ‘fun’ align with and operationalise the ICF framework, helping families to understand the important role they play whilst enabling clinical providers to individualise interventions for each child based on their abilities (Rosenbaum & Gorter 2012). This model is illustrated in Figure 1.

These conceptual shifts have changed how professional services are organised and delivered, reinforcing the central role of family and emphasis on participation (Agarwal, Scher & Tilton 2021; Kim et al. 2021, Rao 2021). This has also aligned with the more vigorous effort to advance evidence-informed therapeutics, leading to the development of standardised guidelines, care pathways and better measures of benefit.

Over time there has been recognition of increasing disparity in uptake at a global level, particularly limited application and adaptation in low-resource settings. Children and their families in these countries are not only at greatest risk but also have the greatest opportunity for impact in adoption of new approaches. Three professional organisations (American Academy of Cerebral Palsy and Developmental Medicine, European Academy of Child Disability and the Australasia Academy of Child Disability) came together to create a global organisation (International Alliance of Academies of Child Disability [IAACD]) that is committed to advance the development of country or regional academies that can better support and build local contextually relevant programmes and services whilst drawing from and contributing to global knowledge developments in the field (Forssberg, Damiano & Armstrong 2022).

In 2014, the Eastern Africa Academy of Child Disability (EAACD) was established and has become a member of the IAACD. The EAACD has held annual professional meetings in Nairobi, Mombasa, Dar es Salaam, Kampala and Addis Ababa. In January 2020, EAACD and IAACD co-hosted a family-centred care workshop in Nairobi with the goal of discussing the 21st-century vision for children with disabilities growing up in East Africa with a specific focus on Kenya. The content and outcome of this workshop are the subject of this report.

## Family-centred care workshop process

**FIGURE 2:**
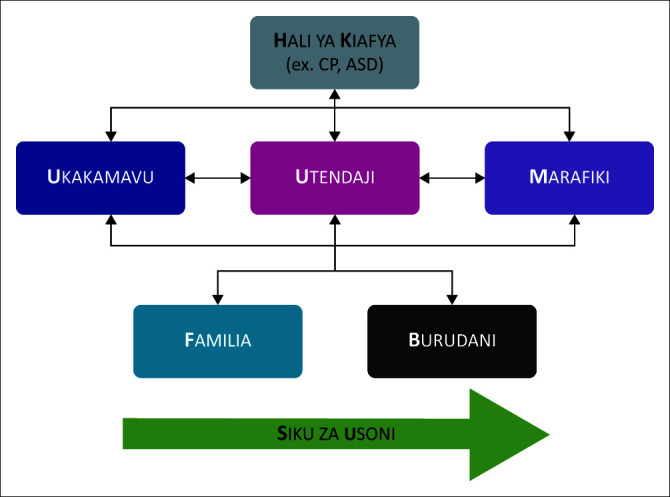
Kiswahili translation of the F-words integrated into the International Classification of Function framework.

The workshop was structured around three thematic areas: (1) advances in understanding of child disability, (2) implementing family-centred care in low-resource settings and (3) planning care across the life course. The workshop used the ‘ICF-F words’ model with Kiswahili translation (Figure 2) in the form of a large poster placed in the meeting room to allow participants visualise and understand the F-Words. This is the first Kiswahili translation of these terms and was made to contextualise the terms for easier assimilation into practice. During a session on the ICF framework and the F-words under the theme ‘Implementing family-centred care in low-resource settings’, the speaker referred to the poster to reinforce the message for the participants. The workshop promoted exploration of local perspectives on disability-related health care practices in East Africa with the goal to make recommendations moving forward on (1) the development of global standards of practice, (2) strategies for dissemination and uptake of evidence-informed protocols and care pathways to guide health care worker practice and (3) strategies to foster more effective networking amongst organisations and promote multidisciplinary practice.

Invitations to the workshop were sent to health professionals, parents of children living with disabilities, parent-run disability organisations and government officials within Kenya, as well as to EAACD members in other Eastern African countries. Participants from IAACD included Peter Rosenbaum, Professor of Paediatrics at McMaster University, an international authority in the field of child disability and the co-creator of the ‘F-Words’, and Arnab Seal, Honorary Senior Lecturer at the University of Leeds and Chair of the Education and Training Committee of IAACD. Over 2 days, 64 participants attended the workshop. Table 1 shows the distribution of participants at the workshop.

**TABLE 1:** Distribution of workshop participants by cadre.

Participant cadre	Number
Government officials (National child and adolescent health unit)	4
Non-governmental organisation representatives	4
Occupational therapists	4
Speech therapist	3
Special education teacher	3
Physiotherapist	4
Clinical psychologist	5
Orthopaedic surgeon	2
Neurosurgeon	1
Parents of children with special needs	5
Paediatric dentist	1
Psychiatrist	2
Medical officer	9
Paediatrician	11
Paediatric neurologist	3
Developmental paediatrician	3

**Total**	**64**

*Source:* Eastern Africa Academy of Childhood Disability (EAACD), *Welcome to EAACD Transforming Eastern Africa*, viewed n.d., from https://www.eaacdafrica.org

The 2-day workshop comprised six sessions, each of which focussed on a unique sub-theme. During each session, four or five multidisciplinary speakers were invited to present on different aspects of the sub-theme. Following these presentations, participants were provided with an opportunity to discuss the sub-theme and presentations in eight round tables groups each comprising eight members and report back on their deliberations to the main group through a rapporteur.

## Workshop outcomes

### Advances in understanding of child disability

There was broad consensus for the value of a 21st-century vision of child disability that was ability and participation focused, although recognition that the concepts articulated still have not yet been fully accepted or integrated into professional practices or into community and government strategies for advancing the cultural and social understanding of disability.

The current vision on child disability encourages governments, organisations and communities to put in place policies and/or processes that facilitate the possibility for young children with disability to have greater participation in society. This approach leads to better inclusivity and advocacy for children with disability and reduces barriers that negate provision for their rights and needs (Leite, Chagas & Rosenbaum 2021). The role of parents and parent advocacy organisations in influencing community and government change was emphasised and working together with health professionals in approaching community or government would be important to advocate for this focus in practice. In the local setting, parent organisations, professional bodies and government agencies were observed to function ‘in silos’, leading to ineffective implementation of policies with potential exclusion of those in greatest need. Educating parents, communities and policymakers on the need to have an ability and participatory approach to child disability was advanced as a way to overcome the existing scenario. The ‘F-Words’ either in English or as translated into Kiswahili were seen as a valuable communication tool, because they would enhance the understanding of a holistic, life course approach to management of disability and inform implementation of habilitation and re-habilitation-based interventions, education and practice (Leite et al. 2021).

### Family-centred care

Practitioners cautioned that the term ‘disability’ as understood in East Africa is problematic because mainly severe forms receive attention. Participants remarked that the medical model remains a dominant force and drives understanding of good health care for children, placing the family in the background and localising the problem to the individual.

The role of the family in care is complex but necessary and transition to a true partnership relationship takes time. Rosenbaum and colleagues embedded the ‘F-words’, including family factors into the ICF framework (Rosenbaum & Gorter 2012) and this has achieved widespread endorsement with translation into 19 different languages. Clinicians remarked that they incorporated components of the F-words framework into practice to varying degrees, highlighting not only the beneficial impact but also challenges because of family reluctance. For example, families would often not allow their child to participate in activities for fear of injury. This requires an active and sensitive negotiation process with the family as the care plan is developed.

Stigma continues to be of concern. Parents and clinicians observed that whilst it is feasible for children with disabilities to have social relationships amongst themselves, it is much harder for them to form relationships with children without disabilities. This compromises the opportunities for participation and may be a common reason for exclusion of children with disabilities from being enrolled in school or fully participating even after enrolment.

A general consensus was that optimal child functioning occurs within a supportive community as described by Ohene, Power and Raghu (2020). Participants remarked that the family input into care was critical; however, the Family Centered Care (FCC) model, which considers family as partners in decision-making, is not encouraged in care locally (Makworo et al. 2016). As a result of a lack of knowledge, data and structural support, participants remarked that it is challenging to incorporate FCC into practice. A system to support FCC collaboration is lacking between policymakers and health professionals and training for healthcare specialists is limited (Makworo et al. 2016).

Other factors encumber FCC implementation including parental commitments – work, caring for others and travelling distance. Participants also highlighted unaddressed mental health problems amongst parents as barriers. Additional difficulties include a need for increased resources, including time required to engage parents because of disconnect in a region where appropriately trained health care workers are limited (Bunning et al. 2020). Structural limitations were also identified, including space, staffing, time restrictions and not having access to multidisciplinary teams. Also, the culture of hospital care was cited because parents are acculturated to believe that care is strictly administered by physicians through hospitals (Makworo et al. 2016).

The benefits to FCC include a platform for supporting families as they work through emotions such as shame, guilt and anxiety (Ohene et al. 2020). The FCC enables families to negotiate within the system, report incidents, provide feedback and create trust between families and care providers (Ohene et al. 2020). In the end, advancing family-centred care provides the child who has a disability with the best opportunity for health development.

### Planning care across the life course

There was general agreement that the lack of country- or community-level data on disability and disability services continues to compromise a 21st-century focus on children with disabilities. Policy documents are fine but without data gathered through health, education and social service systems there is no ability to measure progress, identify areas of need and define inequities in access. This lack of appropriate data was acknowledged by Kenyan government officials attending the workshop and agreed that this should be an area of development.

All children go through transitions and these periods are of special importance to children with disabilities because the access to and nature of services are often tied to specific periods of a child’s life. This is particularly true when children with disabilities turn 18 years of age and transition out of school. Services that were available often end and an increased burden is placed on their families. In the current system, participants remarked that practitioners can improve the quality of transitions through early planning with family using resources available. For example, children known to require assistive devices for mobility would be able to participate much better with early access to wheelchairs. Youth clinics or joint transition clinics were also proposed to affect a smooth transition to adult services. Such clinics are currently not available in East Africa.

## Recommendations

The following recommendations came from a very lively workshop that drew on multiple perspectives in the context of the communities in East Africa:

1. There are significant challenges to implementing culturally sensitive family-centred care in the current context, but individual institutions can use the ‘ICF-F-Words’ framework for moving towards FCC and serving as ‘lead’ agencies where implementation is possible. This might include strengthening existing measures and systems through provision of support such as social workers, facilitation of family participation during care by clinicians and family education beyond treatment.
2. Institutions need to review the capacity and structure of multidisciplinary teams in providing care, exploring strategies for more effective communication, co-location of key disciplines, allowing professionals greater ease of interaction amongst themselves and with families with respect to individual children. Practitioners suggested refinement to an existing system where a family has one booklet where practitioners can add their input, so all care workers and families are aware of the different treatment plans provided. This would help foster continuity of care in lieu of a unified electronic system.
3. Professionals need to more frequently utilise evidence-informed guidelines for assessment and treatment, adjusted as necessary to the context of their community of practice.
4. Pre- and post-employment education reforms need to be explored to ensure professionals are current in their knowledge, practices and attitudes.
5. Government engagement is critical to advancing services for children with disabilities. Professionals need to work with families and family-based organisations to develop a coordinated advocacy strategy to promote government action that is aligned to a national policy framework.
6. Policy makers need to pay attention to development of community and national level data on disability prevalence and the services and programmes available across the life span prioritised.

## Conclusion

The 21st-century understanding of disability creates an expectation to support the development of children with disabilities that is focused on ‘ability’ and participation with assessment, therapeutics and programmes or services that are evidence-informed and advanced in partnership with families. This vision can be achieved in low-resource settings where there is institutional leadership that models best practice and works effectively to influence community and government policy and practices.
